# Do not attempt cardiopulmonary resuscitation (DNACPR) decisions in people admitted with suspected COVID-19: Secondary analysis of the PRIEST observational cohort study

**DOI:** 10.1016/j.resuscitation.2021.04.028

**Published:** 2021-07

**Authors:** Laura Sutton, Steve Goodacre, Ben Thomas, Sarah Connelly

**Affiliations:** School of Health and Related Research (ScHARR), University of Sheffield, Regent Court, Regent Street, Sheffield, S1 4DA, UK

**Keywords:** Do not attempt cardiopulmonary resuscitiation, COVID-19

## Abstract

**Aims:**

We aimed to describe the characteristics and outcomes of adults admitted to hospital with suspected COVID-19 according to their DNACPR decisions, and identify factors associated with DNACPR decisions.

**Methods:**

We undertook a secondary analysis of 13,977 adults admitted to hospital with suspected COVID-19 and included in the Pandemic Respiratory Infection Emergency System Triage (PRIEST) study. We recorded presenting characteristics and outcomes (death or organ support) up to 30 days. We categorised patients as early DNACPR (before or on the day of admission) or late/no DNACPR (no DNACPR or occurring after the day of admission). We undertook descriptive analysis comparing these groups and multivariable analysis to identify independent predictors of early DNACPR.

**Results:**

We excluded 1249 with missing DNACPR data, and identified 3929/12748 (31%) with an early DNACPR decision. They had higher mortality (40.7% v 13.1%) and lower use of any organ support (11.6% v 15.7%), but received a range of organ support interventions, with some being used at rates comparable to those with late or no DNACPR (e.g. non-invasive ventilation 4.4% v 3.5%). On multivariable analysis, older age (p < 0.001), active malignancy (p < 0.001), chronic lung disease (p < 0.001), limited performance status (p < 0.001), and abnormal physiological variables were associated with increased recording of early DNACPR. Asian ethnicity was associated with reduced recording of early DNACPR (p = 0.001).

**Conclusions:**

Early DNACPR decisions were associated with recognised predictors of adverse outcome, and were inversely associated with Asian ethnicity. Most people with an early DNACPR decision survived to 30 days and many received potentially life-saving interventions.

**Registration:**

ISRCTN registry, ISRCTN28342533, http://www.isrctn.com/ISRCTN28342533

## Introduction

In-hospital cardiac arrest is relatively common in patients with COVID-19 and often results in poor outcome. A multicentre cohort study from the United States^1^ reported that 701/5019 (14.0%) critically ill patients with COVID-19 had in-hospital cardiac arrest, with 400/701 (57.1%) receiving CPR, and only 7% of these surviving to hospital discharge with normal or mildly impaired neurological status. Management of cardiac arrest in COVID-19 is further complicated by concerns about infection risk associated with aerosol-generating procedures and consequent risks to staff.^2^

These concerns have raised awareness about the need to consider do not attempt cardiopulmonary resuscitation (DNACPR) decisions when patients are admitted to hospital with suspected COVID-19. An appropriately implemented DNACPR decision can ensure that the patient’s wishes and best interests are addressed, while avoiding futile medical intervention.^3^ However, concerns have been raised about inappropriate use of DNACPR decisions during the pandemic,^4^ leading to the Care Quality Commission being asked to review their use in the United Kingdom (UK).^5^

Previous studies have estimated the prevalence of DNACPR decision in patients admitted to hospital with community-acquired pneumonia^6^, ^7^, ^8^, ^9^, ^10^ and sepsis,^11^, ^12^, ^13^, ^14^ and have attempted to identify factors associated with DNACPR use, but we currently know very little about how DNACPR decisions have been used in people admitted with suspected COVID-19. The Pandemic Respiratory Infection Emergency System Triage (PRIEST) study was established to develop and evaluate triage tools for people presenting to hospital emergency departments with suspected COVID-19.^15^ DNACPR decisions were recorded to facilitate evaluation of triage tools in pre-specified subgroups. We present a post hoc secondary analysis of patients admitted with suspected COVID-19 that aims to describe their characteristics and outcomes according to their DNACPR decision and identify factors associated with recording of a DNACPR decision.

## Methods

PRIEST was an observational cohort study of patients attending an emergency department (ED) in the UK with suspected COVID-19 infection during the first wave of the pandemic. We included patients if the assessing clinician recorded that the patient had suspected COVID-19 in the ED records or completed a standardised assessment form for suspected COVID-19 patients. The clinical diagnostic criteria for COVID-19 during the study were of fever (≥37.8 °C) and at least one of the following respiratory symptoms, which must be of acute onset: persistent cough (with or without sputum), hoarseness, nasal discharge or congestion, shortness of breath, sore throat, wheezing, sneezing. We did not seek consent to collect data but information about the study was provided in the ED and patients could withdraw their data at their request. Patients with multiple presentations to hospital were only included once, using data from the first presentation identified by research staff.

We only included patients who were admitted to hospital after ED assessment because DNACPR planning was considered unlikely to be routinely undertaken for discharged patients and would be limited to a minority of highly selected cases. We also only included adults (age ≥ 16 years) because previous analysis^15^ showed that children with suspected COVID-19 had very low rates of confirmed COVID-19 or adverse outcome.

Baseline characteristics at presentation to the ED were recorded prospectively, using a standardised assessment form that doubled as a clinical record (Appendix 1: Standardised Data Collection Form), or retrospectively, through research staff extracting data onto the standardised form using the clinical records. Research staff collected follow-up data onto a standardised follow-up form (Appendix 2: Follow-up Form) using clinical records up to 30 days after presentation. This included recording whether research staff identified a DNACPR decision made at any time between initial presentation and follow-up, and if so, the date of the decision. We only recorded the first decision and did not record whether it was subsequently changed.

Patients who died or required respiratory, cardiovascular, or renal support were classified as having an adverse outcome. Patients who survived to 30 days without requiring respiratory, cardiovascular, or renal support were classified as having no adverse outcome. Respiratory support was defined as any intervention to protect the patient’s airway or assist their ventilation, including non-invasive ventilation, or acute administration of continuous positive airway pressure. It did not include supplemental oxygen alone or nebulised bronchodilators. Cardiovascular support was defined as any intervention to maintain organ perfusion, such as inotropic drugs, or invasively monitor cardiovascular status, such as central venous pressure or pulmonary artery pressure monitoring, or arterial blood pressure monitoring. We included invasive monitoring as these imply an intention to use cardiovascular support even if it not actually required. It did not include peripheral intravenous cannulation, or fluid administration. Renal support was defined as any intervention to assist renal function, such as haemofiltration, haemodialysis, or peritoneal dialysis. It did not include intravenous fluid administration.

We compared the characteristics and outcomes of those with a DNACPR decision recorded on or before the day of ED assessment (early DNACPR) to those with no DNACPR recorded or a DNACPR decision recorded at a later date (late/no DNACPR). We categorised patients in this way on the assumption that patients with a late DNACPR decision were likely to be systematically different from those with an early decision, with implementation of a late DNACPR decision reflecting the response to intervention. Our categorisation was therefore based on a theoretical framework in which patient characteristics at admission could determine whether a DNACPR decision was recorded at hospital admission, and recording of a DNACPR decision at admission could then determine subsequent use of interventions.

We calculated a National Early Warning Score (2nd version, NEWS2) for adults,^16^ to provide an overall assessment of acute illness severity on a score from zero to 20, based on the first recorded respiratory rate, oxygen saturation, systolic blood pressure, heart rate, level of consciousness and temperature. We calculated a PRIEST COVID-19 clinical severity score, to provide an overall prediction of the risk of adverse outcome on a score from zero to 29, based on NEWS2, age, sex, and performance status.^17^

We also undertook multivariable logistic regression modelling to identify independent predictors of DNACPR decisions. Variables were selected on the basis of clinical interest. Collinearity was observed between Glasgow Coma Scale (GCS) scores and consciousness, recorded as alert, responsive to verbal stimuli, responsive to pain or unconscious (AVPU). Missing AVPU data were imputed using GCS as follows, and GCS dropped from the list of predictors: GCS 15 = Alert, GCS 9–14 = Verbal, GCS 7–8 = Pain, and GCS 3–6 = Unresponsive. Continuous physiological predictors were categorised in accordance with NEWS2 risk categories^16^ where the reference category denotes normal range and increasing category levels indicate increasing deviation from the norm. Data were analysed using SAS v9.4.

## Ethical approval

The North West - Haydock Research Ethics Committee (REC) gave a favourable opinion on the PAINTED study on 25 June 2012 (reference 12/NW/0303) and on the updated PRIEST study on 23rd March 2020. The Confidentiality Advisory Group of the Health Research Authority granted approval to collect data without patient consent in line with Section 251 of the National Health Service Act 2006. The REC approved a substantial amendment to undertake this secondary analysis on 7 January 2021.

## Results

**Fig. 1:**
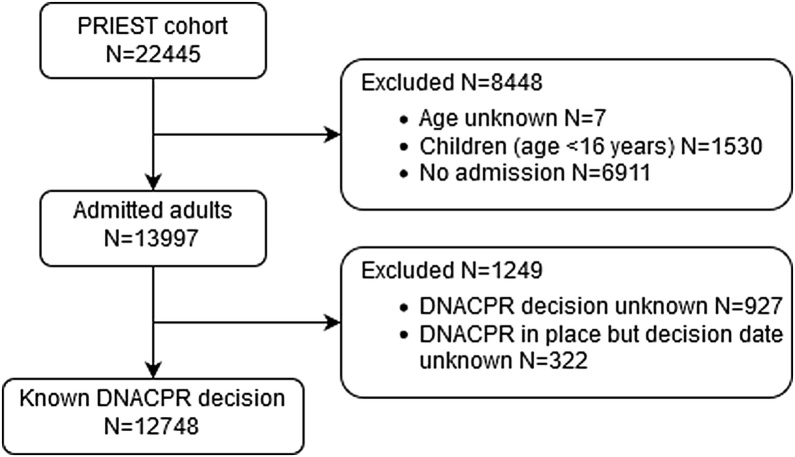
Flow of patients into the analysis.

The PRIEST study recruited 22,484 patients from 70 EDs across 53 sites between 26 March 2020 and 28 May 2020 (22,445 after 39 withdrew their data), including 13,997 adults admitted following ED assessment. Research staff did not complete the DNACPR data item for 1249 patients, so they were excluded from the analysis. The remaining 12,748 were grouped into those with DNACPR decisions made on or before the day of initial ED assessment (N = 3929, 31%) and those with no DNACPR or DNACPR decisions made at a later date (N = 8819, including 7109 with no DNACPR and 1710 late DNACPR). Fig. 1 shows the flow of patients into this analysis.

Table 1 shows presenting characteristics for both groups. Patients with a DNACPR decision recorded on or before their day of attendance tended to be older and have a higher prevalence of comorbidities, limited activity or self-care.

**Table 1:** Presenting characteristics for admitted adults with DNACPR decisions in place by the end of ED assessment (N = 3929) and adults with no DNACPR or DNACPR decision made later (N = 8819).

Characteristic	Statistic/level	Early DNACPR	No/late DNACPR
Age (years)	N	3929	8819
	Mean (SD)	79.4 (10.9)	63.9 (17.6)
	Median (IQR)	81 (73, 87)	65 (52, 78)
Sex	Missing	42	78
	Male	2027 (52.1%)	4704 (53.8%)
	Female	1860 (47.9%)	4037 (46.2%)
Ethnicity	Missing/prefer not to say	601	1746
	UK/Irish/other white	3102 (93.2%)	6027 (85.2%)
	Asian	83 (2.5%)	468 (6.6%)
	Black/African/Caribbean	75 (2.3%)	289 (4.1%)
	Mixed/multiple ethnic groups	27 (0.8%)	102 (1.4%)
	Other	41 (1.2%)	187 (2.6%)
Presenting features	Cough	2117 (53.9%)	5514 (62.5%)
	Shortness of breath	2955 (75.2%)	6678 (75.7%)
	Fever	1777 (45.2%)	4688 (53.2%)
Comorbidities	No Chronic disease	323 (8.2%)	2012 (22.8%)
	Heart Disease	1620 (41.2%)	2036 (23.1%)
	Renal impairment	739 (18.8%)	843 (9.6%)
	Steroid therapy	160 (4.1%)	258 (2.9%)
	Asthma	450 (11.5%)	1446 (16.4%)
	Diabetes	1123 (28.6%)	2034 (23.1%)
	Active malignancy	370 (9.4%)	508 (5.8%)
	Immunosuppression	126 (3.2%)	326 (3.7%)
	Other chronic lung disease	1219 (31%)	1685 (19.1%)
	Hypertension	1721 (43.8%)	3039 (34.5%)
Symptom duration (days)	N	3396	7986
	Mean (SD)	5.7 (6.8)	7.5 (8.4)
	Median (IQR)	3 (1, 7)	5 (2, 10)
Heart rate (beats/min)	N	3867	8664
	Mean (SD)	95.5 (23)	97.4 (22)
	Median (IQR)	93 (80, 109)	96 (82, 111)
Respiratory rate (breaths/min)	N	3867	8625
	Mean (SD)	25.5 (7.5)	23.9 (6.9)
	Median (IQR)	24 (20, 29)	22 (19, 28)
Systolic BP (mmHg)	N	3834	8629
	Mean (SD)	131.8 (27.8)	134.1 (25.6)
	Median (IQR)	130 (112, 149)	132 (117, 149)
Diastolic BP (mmHg)	N	3813	8587
	Mean (SD)	73.5 (17.1)	77.4 (16.3)
	Median (IQR)	72 (62, 84)	77 (67, 87)
Temperature (°C)	N	3803	8576
	Mean (SD)	37.1 (1.2)	37.3 (1.2)
	Median (IQR)	37 (36.4, 37.9)	37.2 (36.5, 38.1)
Oxygen saturation (%)	N	3893	8741
	Mean (SD)	92.6 (8)	94.1 (6.8)
	Median (IQR)	95 (91, 97)	96 (93, 98)
Glasgow Coma Scale	N	2985	6643
	Mean (SD)	13.9 (2.1)	14.7 (1.3)
	Median (IQR)	15 (14, 15)	15 (15, 15)
AVPU	Missing	610	1009
	Alert	2877 (86.7%)	7486 (95.9%)
	Verbal	291 (8.8%)	229 (2.9%)
	Pain	97 (2.9%)	50 (0.6%)
	Unresponsive	54 (1.6%)	45 (0.6%)
Performance status	Missing	222	455
	Unrestricted normal activity	453 (12.2%)	4341 (51.9%)
	Limited strenuous activity, can do light activity	448 (12.1%)	1160 (13.9%)
	Limited activity, can self care	923 (24.9%)	1351 (16.2%)
	Limited self care	1153 (31.1%)	993 (11.9%)
	Bed/chair bound, no self care	730 (19.7%)	519 (6.2%)
NEWS2 score	N	3911	8723
	Mean (SD)	6.4 (3.3)	5.1 (3.1)
	Median (IQR)	6 (4, 9)	5 (3, 7)
PRIEST score	N	3870	8645
	Mean (SD)	12.5 (3.9)	8.9 (4.1)
	Median (IQR)	12 (10, 15)	9 (6, 12)

**Fig. 2:**
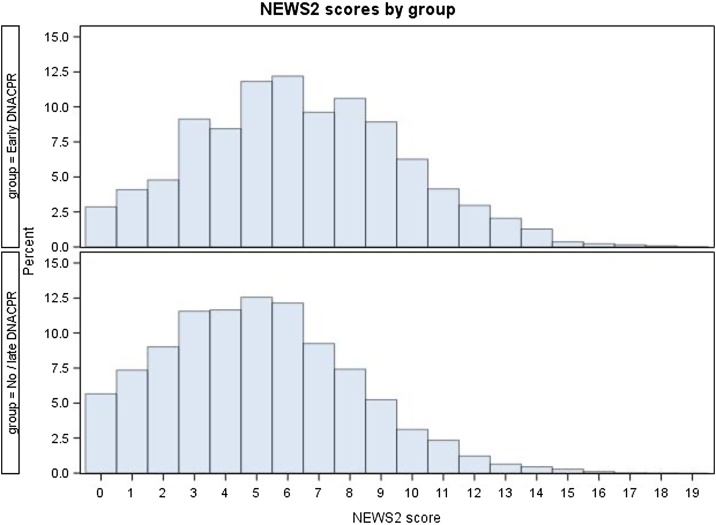
NEWS2 score distribution by DNACPR status.

**Fig. 3:**
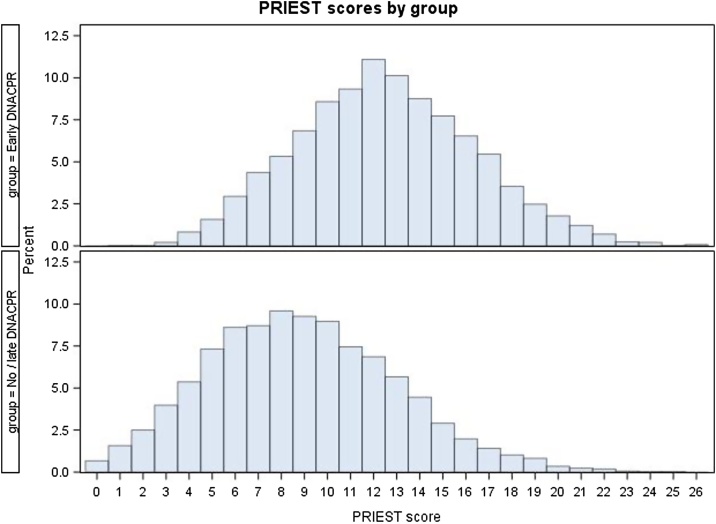
PRIEST score distribution by DNACPR status.

Fig. 2 compares the NEWS2 scores for the two groups and shows that those with early DNACPR decisions tended to be more acutely unwell (median score 6 versus 5). Fig. 3 compares the PRIEST COVID-19 clinical severity scores for the two groups and shows that those with early DNACPR decisions were at a higher risk of adverse outcome (median score 12 versus 9, respectively indicating 38% versus 26% expected risk of a 30-day adverse outcome).^17^

Table 2 gives location of admission, pathogen confirmation and adverse outcome data for the two groups. Patients with an early DNACPR decision had a higher mortality rate but most (59.4%) survived to 30 days. They also had lower use of critical care and organ support, but a significant proportion (11.6%) received organ support. Table 2 shows the highest level of organ support received, according to a predefined hierarchy (corresponding to the order presented in the table). Patients with early DNACPR decisions received a wide range of interventions, some at comparable rates to those with no or a late DNACPR decision (e.g. non-invasive ventilation and high flow nasal oxygen).

**Table 2:** Outcome data for admitted adults with DNACPR decisions in place by the end of ED assessment (N = 3929) and adults with no DNACPR or DNACPR decision made later (N = 8819).

Outcome	Level	Early DNACPR	No/late DNACPR
Location of first admission	Missing	98	194
	Ward	3717 (97%)	7802 (90.5%)
	ITU	57 (1.5%)	667 (7.7%)
	HDU	57 (1.5%)	156 (1.8%)
Respiratory pathogen	COVID-19	1791 (45.6%)	3367 (38.2%)
	Influenza	6 (0.2%)	14 (0.2%)
	Other	235 (6%)	701 (7.9%)
	None identified	1897 (48.3%)	4737 (53.7%)
Mortality status	Missing	0	0
	Alive	2328 (59.3%)	7668 (86.9%)
	Dead	1601 (40.7%)	1151 (13.1%)
	Death with organ support	251 (15.7%)	373 (32.4%)
	Death without organ support	1350 (84.3%)	778 (67.6%)
Organ support	Respiratory	423 (10.8%)	1313 (14.9%)
	Mechanical ventilation	51 (1.3%)	509 (5.8%)
	Non-invasive ventilation	173 (4.4%)	292 (3.3%)
	Continuous positive airway pressure	125 (3.2%)	386 (4.4%)
	High-flow nasal oxygen	74 (1.9%)	126 (1.4%)
	Cardiovascular	47 (1.2%)	426 (4.8%)
	Extracorporeal membrane oxygenation	0 (0%)	13 (0.1%)
	Inotropic/vasopressor drugs	29 (0.7%)	283 (3.2%)
	Central venous pressure measurement	2 (0.1%)	25 (0.3%)
	Intra-arterial BP measurement	16 (0.4%)	105 (1.2%)
	Renal	29 (0.7%)	172 (2%)
	Haemofiltration	7 (0.2%)	93 (1.1%)
	Haemodialysis	22 (0.6%)	75 (0.9%)
	Peritoneal dialysis	0 (0%)	4 (0%)
	Any	455 (11.6%)	1386 (15.7%)

Table 3 shows the results of the multivariable logistic regression model. Older age, active malignancy, chronic lung disease, limited performance status, abnormal respiratory rate, lower oxygen saturation, and lower alertness were all associated with increased use of early DNACPR. Asian ethnicity was associated with a lower use of early DNACPR.

**Table 3:** Multivariable analysis of predictors of early DNACPR use.

Effect	Odds ratio	95% CI	p-Value
Age	1.054	(1.049, 1.060)	<0.001
Male sex	1.010	(0.905, 1.128)	0.859
Ethnicity (ref = UK/Irish/other white)			
Asian	0.571	(0.416, 0.783)	0.001
Black/African/Caribbean	0.730	(0.524, 1.017)	0.063
Mixed/multiple ethnic groups	0.993	(0.568, 1.737)	0.982
Other	0.647	(0.410, 1.020)	0.061
Shortness of breath	1.150	(1.005, 1.316)	0.042
Cough	1.012	(0.903, 1.133)	0.841
Fever	0.954	(0.851, 1.069)	0.415
No chronic disease	0.753	(0.609, 0.931)	0.009
Heart disease	1.182	(1.048, 1.333)	0.006
Renal impairment	1.241	(1.067, 1.445)	0.005
Steroid therapy	1.268	(0.971, 1.657)	0.082
Asthma	0.900	(0.765, 1.060)	0.209
Diabetes	1.120	(0.987, 1.271)	0.080
Active malignancy	1.604	(1.319, 1.951)	<0.001
Immunosuppression	1.117	(0.835, 1.494)	0.455
Other chronic lung disease	1.456	(1.280, 1.656)	<0.001
Hypertension	0.883	(0.786, 0.993)	0.038
Symptom duration (days)	0.993	(0.986, 1.001)	0.076
Pulse rate (beats/min; ref = 51−90)			
41−50 or 91−110	1.121	(0.987, 1.274)	0.079
111−130	1.095	(0.929, 1.290)	0.280
≤40 or ≥131	1.030	(0.816, 1.300)	0.802
Respiratory rate (breaths/min; ref = 12−20)			
9−11	0.772	(0.115, 5.173)	0.790
21−24	1.175	(1.019, 1.355)	0.027
≤8 or ≥25	1.361	(1.187, 1.560)	<0.001
Systolic BP (mmHg; ref = 111−219)			
101−110	1.164	(0.968, 1.400)	0.107
91−100	1.347	(1.066, 1.703)	0.013
≤90 or ≥220	1.177	(0.891, 1.555)	0.251
Temperature (°C; ref = 36.1−38.0)			
35.1−36.0 or 38.1−39.0	0.916	(0.807, 1.039)	0.173
≥39.1	0.885	(0.693, 1.131)	0.330
≤35.0	1.085	(0.764, 1.540)	0.650
Oxygen saturation (%; ref=≥96)			
94−95	1.041	(0.898, 1.206)	0.595
92−93	1.127	(0.938, 1.354)	0.203
≤91	1.329	(1.153, 1.532)	<0.001
AVPU (ref = Alert)			
Verbal	1.905	(1.563, 2.323)	<0.001
Pain	2.651	(1.644, 4.272)	<0.001
Unresponsive	2.620	(1.429, 4.803)	0.002
Performance status (ref = Unrestricted normal activity)			
Limited strenuous activity, can do light activity	1.885	(1.565, 2.269)	<0.001
Limited activity, can self care	2.629	(2.222, 3.110)	<0.001
Limited self care	4.100	(3.448, 4.876)	<0.001
Bed/chair bound, no self care	5.437	(4.438, 6.660)	<0.001

Finally, we undertook a post hoc sensitivity analysis to determine whether the multivariable analysis was robust to using a more liberal definition of early DNACPR, in which 870 patients with a DNACPR decision on the day after attendance were reclassified as having an early decision. This is presented in the supplementary table and shows that the most significant predictors were unchanged.

## Discussion

We found that 31% of adults admitted to hospital with suspected COVID-19 during the first phase of the pandemic had a DNACPR decision recorded on or before their day of attendance, after excluding those who could not be classified. Most patients (59.4%) with an early DNACPR decision survived to 30 days and 11.6% received some form of organ support. These findings show that potentially life-saving treatments were provided to a significant proportion of people, potentially addressing concerns that DNACPR decisions may be conflated with ‘do not provide active treatment’.^18^ The use of invasive intervention, particularly mechanical ventilation, in people with a DNACPR decision was an unexpected finding. Contact with participating site investigators suggested that this could be explained by use of the ReSPECT process in discussions about resuscitation, in which the patient is encouraged to explicitly indicate which treatments they want in a future situation where they are unable to make or express choices.^19^ The ReSPECT process therefore allows patients to consent to mechanical ventilation but decline cardiopulmonary resuscitation if it is subsequently required.

Older age, active malignancy, chronic lung disease (excluding asthma), and lower performance status were associated with increased use of early DNACPR, whereas Asian ethnicity was associated with decreased use. Patients with early DNACPR decisions tended to be more acutely ill, with higher NEWS2 scores. Abnormal respiratory rate, lower oxygen saturation, and lower alertness were associated with increased use of early DNACPR.

Our findings suggest a higher rate of DNACPR use (31%) than identified in previous studies of other respiratory conditions or sepsis. Studies of patients admitted with community-acquired pneumonia reported rates of DNACPR use ranging from 13% to 29%,^6^, ^7^, ^8^, ^9^, ^10^ while studies in severe sepsis or septic shock reported rates ranging from 9% to 20%.^11^, ^12^, ^13^, ^14^ DNACPR decisions in these studies were associated with older age, but conflicting findings were reported around the use of invasive procedures. Sakari et al.^11^ and Bradford et al.^12^ reported that DNACPR decision were associated with lower rates of invasive procedures, while Powell et al.^13^ reported no difference, and Huang et al.^14^ reported a higher rate of arterial or central venous cannulation in those with a DNACPR decision. Our data cannot determine why there was a relatively high rate of DNACPR use, so further research could be helpful to identify whether this reflects concerns around personal risks to staff or the lack of effective treatments in the first phase of the pandemic.

We found an association between Asian ethnicity and decreased use of early DNACPR decisions compared to White ethnicity. The odds ratio for Black/African/Caribbean ethnicity also suggested decreased use but was not significant. Previous studies from the United States have shown less use of DNACPR decisions among African-American, Asian, and Hispanic patients,^20^, ^21^, ^22^ and Black patients tend to receive more life-prolonging treatment at end of life care.^23^ A systematic review of end of life decisions for people from ethnic minority groups suggested that Hispanic and African American people had advance care plans documented less often, citing religious coping and spirituality as factors.^24^ A scoping review of culturally- and spiritually-sensitive end-of-life care highlighted a multitude of factors influencing end-of-life care and subsequent experiences by culturally- and spiritually-diverse groups.^25^ Further research of DNACPR decisions in relation to ethnicity is clearly required.

Studies of DNACPR use in COVID-19 are currently limited. Alhatem et al.^26^ analysed 1270 patients admitted across two hospitals with COVID-19, of whom 750/1270 (59%) had a DNACPR decision at admission, and 570/750 (76%) of these died. Age over 60, male sex, and comorbidities were associated with DNACPR at admission. Coleman et al.^27^ examined records of DNACPR decisions at a single centre from 2017 to 2020 and showed an increased rate of DNACPR use during pandemic, with patients tending to be younger and have fewer comorbidities. It is unclear whether these findings reflect an increased overall need for DNACPR decisions during the pandemic or increased willingness to use DNACPR decisions in COVID-19.

This study was based on a large representative sample of adults admitted with suspected COVID-19, but has a number of limitations. DNACPR decisions were recorded to facilitate subgroup analyses addressing the primary purpose of the study rather than addressing the aims of this secondary analysis. We were unable to include 1249/13997 (9%) cases because data were missing or uncertain regarding the use or timing for DNACPR. Recording of DNACPR decision in the notes was inconsistent between sites and some recorded decisions may have been missed. Our categorisation on the basis of timing of DNACPR decision and assumption that later DNACPR decisions are qualitatively different to early decisions could be challenged, and the categorisation may have been too early to capture the impact of senior or specialist review. We did not collect any detailed data to allow us to explore the reasons behind DNACPR decisions, so we are unable to offer explanations for the associations identified in our analysis. We also did not collect data on whether sites used ReSPECT or other similar processes, or isolated DNACPR decisions. Our suggestion that the ReSPECT process could explain the use of invasive interventions in people with a DNACPR decision is based on informal contacts and requires further research. The use of the ReSPECT process could also undermine our rationale for categorising DNACPR decisions as early versus late or no decision, and suggests a complex relationship between DNACPR decisions and subsequent interventions.

## Conclusion

We found that many patients with an early DNACPR decision went on to receive life-saving interventions and most survived to 30 days. Early DNACPR decisions were associated with older age, lower performance status, active malignancy, chronic lung disease, and severe illness, as indicated by physiological parameters. We found some evidence of an association between ethnicity and DNACPR decisions that requires further research.

## Role of the funding source

The PRIEST study was funded by the United Kingdom National Institute for Health Research Health Technology Assessment(HTA) programme (project reference 11/46/07). The funder played no role in the study design; in the collection, analysis, and interpretation of data; in the writing of the report; and in the decision to submit the article for publication. The views expressed are those of the authors and not necessarily those of the NHS, the NIHR or the Department of Health and Social Care.

## Credit author statement

**Steve Goodacre:** conceived and designed the study. **Ben Thomas:** oversaw data acquisition. **Laura Sutton:** analysed the data. **Sarah Connelly:** undertook literature searches. **Steve Goodacre, Laura Sutton**, **Ben Thomas** and **Sarah Connelly** interpreted the data. All authors contributed to drafting the manuscript. **Steve Goodacre** is the guarantor of the paper. The corresponding author attests that all listed authors meet authorship criteria and that no others meeting the criteria have been omitted.

## Conflict of interest statement

All authors have completed the ICMJE uniform disclosure form at www.icmje.org/coi_disclosure.pdf and declare: grant funding to their employing institutions from the National Institute for Health Research; no financial relationships with any organisations that might have an interest in the submitted work in the previous three years; no other relationships or activities that could appear to have influenced the submitted work.

## Data sharing

Anonymised data are available from the corresponding author upon reasonable request (contact details on first page).
