# Supplementary material for: Do not attempt cardiopulmonary resuscitation (DNACPR) decisions in people admitted with suspected COVID-19: Secondary analysis of the PRIEST observational cohort study
Source: Resuscitation. 2021 Jul;164:130–8. doi: 10.1016/j.resuscitation.2021.04.028 (PMC8095017; doi:10.1016/j.resuscitation.2021.04.028)
Supplement: Supplementary file 6 [file mmc6.docx]

**Appendix 6: Supporting Research Staff**

Marie Hyslop

Dan Beever

Samuel Keating

Kerry Wilson

Heather Dakin

Edwin Burkinshaw

Kirsty Pemberton

Tim Chater

Chris Turtle

Emily Turton

Matthew Bursnall

Mike Bradburn

Jennifer Petrie

Lizzie Swaby

Gemma Hackney

Judith Cohen
