# Supplementary material for: Do not attempt cardiopulmonary resuscitation (DNACPR) decisions in people admitted with suspected COVID-19: Secondary analysis of the PRIEST observational cohort study
Source: Resuscitation. 2021 Jul;164:130–8. doi: 10.1016/j.resuscitation.2021.04.028 (PMC8095017; doi:10.1016/j.resuscitation.2021.04.028)
Supplement: Supplementary file 7 [file mmc7.docx]

## Supplementary table: Comparison of the original multivariable analysis to a sensitivity analysis with 870 late DNACPR cases reclassified as early DNACPR

|  | **ORIGINAL** | | | | **SENSITIVITY** | | |
| --- | --- | --- | --- | --- | --- | --- | --- |
| **Effect** | **Odds ratio** | **95% CI** | **p-value** | **Odds ratio** | | **95% CI** | **p-value** |
| Age | 1.054 | (1.049, 1.060) | <0.001 | 1.060 | | (1.055, 1.066) | <0.001 |
| Male sex | 1.010 | (0.905, 1.128) | 0.859 | 1.013 | | (0.907, 1.130) | 0.823 |
| Ethnicity (ref=UK/Irish/other white) |  |  |  |  | |  |  |
| Asian | 0.571 | (0.416, 0.783) | 0.001 | 0.587 | | (0.435, 0.792) | 0.001 |
| Black/African/Caribbean | 0.730 | (0.524, 1.017) | 0.063 | 0.721 | | (0.524, 0.993) | 0.046 |
| Mixed/multiple ethnic groups | 0.993 | (0.568, 1.737) | 0.982 | 0.974 | | (0.569, 1.670) | 0.925 |
| Other | 0.647 | (0.410, 1.020) | 0.061 | 0.767 | | (0.496, 1.186) | 0.233 |
| Shortness of breath | 1.150 | (1.005, 1.316) | 0.042 | 1.111 | | (0.972, 1.27) | 0.122 |
| Cough | 1.012 | (0.903, 1.133) | 0.841 | 0.990 | | (0.884, 1.109) | 0.862 |
| Fever | 0.954 | (0.851, 1.069) | 0.415 | 0.959 | | (0.857, 1.074) | 0.472 |
| No chronic disease | 0.753 | (0.609, 0.931) | 0.009 | 0.778 | | (0.634, 0.954) | 0.016 |
| Heart disease | 1.182 | (1.048, 1.333) | 0.006 | 1.212 | | (1.074, 1.368) | 0.002 |
| Renal impairment | 1.241 | (1.067, 1.445) | 0.005 | 1.312 | | (1.123, 1.532) | 0.001 |
| Steroid therapy | 1.268 | (0.971, 1.657) | 0.082 | 1.269 | | (0.969, 1.663) | 0.084 |
| Asthma | 0.900 | (0.765, 1.060) | 0.209 | 0.876 | | (0.747, 1.028) | 0.106 |
| Diabetes | 1.120 | (0.987, 1.271) | 0.080 | 1.138 | | (1.003, 1.292) | 0.045 |
| Active malignancy | 1.604 | (1.319, 1.951) | <0.001 | 1.871 | | (1.532, 2.285) | <0.001 |
| Immunosuppression | 1.117 | (0.835, 1.494) | 0.455 | 1.032 | | (0.776, 1.373) | 0.828 |
| Other chronic lung disease | 1.456 | (1.280, 1.656) | <0.001 | 1.505 | | (1.323, 1.713) | <0.001 |
| Hypertension | 0.883 | (0.786, 0.993) | 0.038 | 0.905 | | (0.805, 1.017) | 0.094 |
| Symptom duration (days) | 0.993 | (0.986, 1.001) | 0.076 | 0.993 | | (0.986, 1.000) | 0.058 |
| Pulse rate (beats/min; ref=51-90) |  |  |  |  | |  |  |
| 41-50 or 91-110 | 1.121 | (0.987, 1.274) | 0.079 | 1.097 | | (0.966, 1.246) | 0.153 |
| 111-130 | 1.095 | (0.929, 1.290) | 0.280 | 1.086 | | (0.923, 1.278) | 0.321 |
| ≤40 or ≥131 | 1.030 | (0.816, 1.300) | 0.802 | 0.989 | | (0.785, 1.246) | 0.927 |
| Respiratory rate (breaths/min; ref=12-20) |  |  |  |  | |  |  |
| 9-11 | 0.772 | (0.115, 5.173) | 0.790 | 1.198 | | (0.181, 7.941) | 0.852 |
| 21-24 | 1.175 | (1.019, 1.355) | 0.027 | 1.274 | | (1.107, 1.466) | 0.001 |
| ≤8 or ≥25 | 1.361 | (1.187, 1.560) | <0.001 | 1.446 | | (1.263, 1.656) | <0.001 |
| Systolic BP (mmHg; ref=111-219) |  |  |  |  | |  |  |
| 101-110 | 1.164 | (0.968, 1.400) | 0.107 | 1.321 | | (1.098, 1.589) | 0.003 |
| 91-100 | 1.347 | (1.066, 1.703) | 0.013 | 1.525 | | (1.202, 1.934) | 0.001 |
| ≤90 or ≥220 | 1.177 | (0.891, 1.555) | 0.251 | 1.290 | | (0.967, 1.721) | 0.084 |
| Temperature (°C; ref=36.1-38.0) |  |  |  |  | |  |  |
| 35.1-36.0 or 38.1-39.0 | 0.916 | (0.807, 1.039) | 0.173 | 0.945 | | (0.833, 1.071) | 0.375 |
| ≥39.1 | 0.885 | (0.693, 1.131) | 0.330 | 0.889 | | (0.700, 1.129) | 0.336 |
| ≤35.0 | 1.085 | (0.764, 1.540) | 0.650 | 1.279 | | (0.889, 1.841) | 0.185 |
| Oxygen saturation (%; ref=≥96) |  |  |  |  | |  |  |
| 94-95 | 1.041 | (0.898, 1.206) | 0.595 | 0.991 | | (0.857, 1.146) | 0.903 |
| 92-93 | 1.127 | (0.938, 1.354) | 0.203 | 1.115 | | (0.930, 1.338) | 0.240 |
| ≤91 | 1.329 | (1.153, 1.532) | <0.001 | 1.344 | | (1.165, 1.549) | <0.001 |
| AVPU (ref=Alert) |  |  |  |  | |  |  |
| Verbal | 1.905 | (1.563, 2.323) | <0.001 | 1.801 | | (1.459, 2.223) | <0.001 |
| Pain | 2.651 | (1.644, 4.272) | <0.001 | 3.048 | | (1.767, 5.259) | <0.001 |
| Unresponsive | 2.620 | (1.429, 4.803) | 0.002 | 1.716 | | (0.915, 3.217) | 0.092 |
| Performance status (ref=Unrestricted normal activity) |  |  |  |  | |  |  |
| Limited strenuous activity, can do light activity | 1.885 | (1.565, 2.269) | <0.001 | 1.855 | | (1.560, 2.206) | <0.001 |
| Limited activity, can self care | 2.629 | (2.222, 3.110) | <0.001 | 2.692 | | (2.297, 3.154) | <0.001 |
| Limited self care | 4.100 | (3.448, 4.876) | <0.001 | 4.392 | | (3.714, 5.193) | <0.001 |
| Bed/chair bound, no self care | 5.437 | (4.438, 6.660) | <0.001 | 6.101 | | (4.976, 7.481) | <0.001 |
